# Automatic rating of therapist facilitative interpersonal skills in text: A natural language processing application

**DOI:** 10.3389/fdgth.2022.917918

**Published:** 2022-08-16

**Authors:** James M. Zech, Robert Steele, Victoria K. Foley, Thomas D. Hull

**Affiliations:** ^1^ Department of Counseling and Clinical Psychology, Teachers College, Columbia University, New York, NY, United States; ^2^ Department of Research & Development, Talkspace, New York, NY, United States; ^3^ Department of Computer Science, The University of Southern California, Los Angeles, CA, United States; ^4^ Department of Psychology, The New School for Social Research, New York, NY, United States

**Keywords:** Facilitative interpersonal skills, common factors, messaging therapy, machine learning, BERT

## Abstract

**Background:**

While message-based therapy has been shown to be effective in treating a range of mood disorders, it is critical to ensure that providers are meeting a consistently high standard of care over this medium. One recently developed measure of messaging quality–The Facilitative Interpersonal Skills Task for Text (FIS-T)–provides estimates of therapists’ demonstrated ability to convey psychotherapy's common factors (e.g., hopefulness, warmth, persuasiveness) over text. However, the FIS-T's scoring procedure relies on trained human coders to manually code responses, thereby rendering the FIS-T an unscalable quality control tool for large messaging therapy platforms.

**Objective:**

In the present study, researchers developed two algorithms to automatically score therapist performance on the FIS-T task.

**Methods:**

The FIS-T was administered to 978 messaging therapists, whose responses were then manually scored by a trained team of raters. Two machine learning algorithms were then trained on task-taker messages and coder scores: a support vector regressor (SVR) and a transformer-based neural network (DistilBERT).

**Results:**

The DistilBERT model had superior performance on the prediction task while providing a distribution of ratings that was more closely aligned with those of human raters, versus SVR. Specifically, the DistilBERT model was able to explain 58.8% of the variance (*R*^2 ^= 0.588) in human-derived ratings and realized a prediction mean absolute error of 0.134 on a 1–5 scale.

**Conclusions:**

Algorithms can be effectively used to ensure that digital providers meet a consistently high standard of interactions in the course of messaging therapy. Natural language processing can be applied to develop new quality assurance systems in message-based digital psychotherapy.

## Introduction

Even before the widespread adoption of telehealth practices in response to the COVID-19 pandemic, clinicians and researchers alike have had to grapple with the promise and implications of digitally-delivered psychotherapy. In light of a growing and intractable mental health crisis, technologically-enabled interventions can help to lower the financial and geographic barriers to therapy, ultimately leading to a more equitable and scalable suite of interventions (1, 2). Of the many digital psychotherapy interventions developed in the recent past, messaging therapy stands out as a particularly scalable intervention. In messaging therapy, client and therapist communicate through text, writing to each other over either a mobile app or email. This relatively asynchronous form of communication allows for greater flexibility in scheduling for both parties and lowers other otherwise necessary treatment costs (e.g., travel, office space). Recent findings from open trials suggest that messaging therapy may be effective for treatment-seeking populations experiencing depression and anxiety (3, 4) and trauma (5). Yet while messaging therapy may be broadly effective and lowers structural barriers to mental health treatment, its rising popularity poses a new challenge: treatment quality assurance. To meet these challenges, it may be possible to leverage new tools and methodologies that can allow us to evaluate therapeutic interactions in high resolution, rather than rely solely on batteries of self-report measures (6). One promising emerging avenue of research is the application of natural language processing (NLP) methods to therapeutic conversations. NLP methods are uniquely well-suited to digital treatment quality assurance for several reasons. First, NLP requires transcripts of psychotherapy conversations, the creation of which is technically simple in digital therapy but can be prohibitively time- and cost-intensive in traditional research contexts where psychotherapy is delivered face-to-face. Further, NLP methods tend to perform exceptionally well given large conversational datasets, which digital telemental health providers are easily able to generate over the course of regular care.

When searching for messaging therapy's mechanisms of action using NLP, it may be just as fruitful to search for markers of effective *providers* versus markers of effective *treatments*. Clinical science has produced decades of research on features of effective mental health treatments, yet relatively little researcher attention has been given to the markers of effective providers*.* These therapist effects have been found to account for an estimated 3–17% of the variance in therapy outcomes (7–9), with higher therapist effects found in more naturalistic settings (10). In one study, the top 10% of therapists realized 2–3 times as much patient change in pre-post outcomes as their bottom 10% counterparts, controlling for client severity (7). Another study of 118 therapists carried out between 2000 and 2009 found that the patients of top performing therapists had almost twice the recovery rates (76%) as those of the lowest performing therapists (40%) (11).

Several markers of effective therapists have been proposed, including the ability to inspire hopefulness (12), maintain therapeutic presence (13), demonstrate appropriate responsiveness (14), or hold the patient in unconditional positive regard (15). These therapist effects fall into the category of nonspecific or common factors that play a therapeutic role across all psychosocial treatments (16, 17). Several of these common factors have been operationalized and measured *via* the Facilitative Interpersonal Skills Behavioral Task and Coding System (FIS) (18). In the original FIS Task, participants are presented with video prompts (“vignettes”) of challenging psychotherapy patients or situations and are instructed to respond to each vignette as if they were providing therapy to the mock patient. Therapist responses are then rated by observers along eight clinical domains: verbal fluency (VF), hope & positive expectations (HPE), persuasiveness (PER), emotional expression (EEx), warmth, acceptance, & understanding (WAU), empathy (EMP), alliance bond capacity (ABC), and alliance rupture-repair responsiveness (ARRR). A task-taker's global FIS score can then be computed by averaging a participant's scores across all eight domains.

Prior research has found that therapists' performance on the FIS Task is predictive of their effectiveness. In one study, the FIS was administered to 25 therapists who had previously treated 1,141 patients and found that therapists with higher FIS yielded greater rates of patient improvement than therapists with lower FIS (18). A follow-up study then found greater patient 12-session symptom reduction among therapists who scored higher on the FIS Task (19). This study also found the FIS Task to be a reliable predictor of provider effectiveness for both therapist-trainees and non-therapists providing emotional support.

While this body of work has shown the FIS Task to be a robust predictor of clinical competency (20), there are two key barriers that prevent the FIS Task from being implemented at scale in digital care environments. First, the task was developed to assess in-person communication patterns, not communication by texting. Although messaging platforms offer video-based therapy, many clients opt for a purely message-based treatment paradigm wherein all patient-provider communication is facilitated over SMS messaging (4). To capture FIS skills as conveyed over text messaging, the FIS Task must be translated into a text-based format. Accordingly, researchers have recently developed a revised FIS Performance Task (FIS-T) that adapts the video FIS to a messaging context (21).

The second major implementation challenge lies in the video FIS Task's reliance on trained human observers, each of whom must manually rate each participant response on multiple dimensions. Given the time-intensive nature of this training and scoring process, administering the FIS-T to a large therapist network with thousands of providers and returning reliable scores would be a prohibitively costly and time-intensive undertaking. Yet advances in machine learning and natural language processing (NLP) techniques allow the FIS-T to be implemented at scale for large therapist network evaluation and training. In the last decade, researchers have explored a variety of machine learning applications to psychotherapy process-outcome research with reasonable success (22, 23). Applying NLP to the problem of behavioral task scoring stands as one of the most feasible and high-value applications at the intersection of machine learning and psychotherapy, principally because human raters and computers are exposed to the same data used to generate scores (24).

In the present study, we built on this work by developing two algorithms to score providers’ responses to the FIS-T Task for messaging therapy. Our first goal was to build a model that could provide reliable response ratings that were commensurate with those of human raters. Relatedly, we sought to identify the linguistic features that distinguished high-scoring FIS-T responses from low-scoring responses.

## Method

### Dataset development

The dataset for the present study is derived from an archival dataset that was generated as part of the routine onboarding process for messaging therapy providers on a digital mental health platform (Talkspace.com). As part of this routine procedure, four graduate-level coders were trained to rate therapists’ open-ended responses to the FIS-T Task stimuli until they demonstrated excellent inter-rater reliability (average two-way random effects ICC = 0.92). Afterwards, a total of 978 digital messaging therapists completed the FIS-T task between March and June of 2020. Each participating therapist responded to the FIS-T Task and then had their responses scored across 8 FIS-T skill domains by the trained four graduate-level coders using the adapted FIS-T Observer Rating Manual (see Table 1). Scores were then verified by a graduate-level coding manager as a means of ensuring broad inter-rater agreement. These final scores for the 978 therapists were then used to train our automatic scoring algorithm. Crucially, each therapist's FIS-T task performance was only scored by a single rater, which was later verified by a coding supervisor.

**Table 1:** FIST domain descriptions.

FIS-T Domain	Description	Illustrative Text Subset
Medium Sensitivity (MS)	Employing communication strategies and a conversational style that translates well in the context of messaging therapy. Examples of effective strategies include paralinguistic restitution and appropriate use of spelling, punctuation, and grammar, and emojis to convey meaning.	* … reading your last text just sent a shiver down my spine. That must have been an uncomfortable experience :( … *
Hope & Positive Expectations (HPE)	Communicating that facilitates a client's agency and self-efficacy through hope, realistic optimism, and positive expectations regarding the client's ability to change and reach their goals.	* … That's fantastic news! Yes, it's a tight deadline, but you’ve made those before … you got this … *
Persuasiveness (PER)	Clearly and convincingly conveying adaptive views and reappraisals which may be different from those communicated by the client. Persuasive messages convey confidence, certainty, and authority while reframing a client's experiences.	* … I really admire how you’ve been putting your all into the job search. That alone says a lot about the person you are and the future you’re creating … *
Emotional Engagement (EEn)	Conveying emotional investment through text and using communication strategies that elicit emotional engagement on the part of the client.	* … That must have been extremely difficult in the moment… do you remember how you felt in your body?…*
Warmth, Acceptance, & Understanding (WAU)	The demonstrated capacity to care for and accept the client. Attitudes which indicate an absence of acceptance include a judgmental tone, condescension, or exasperation, whereas acceptance is indicated by a caring attitude and determination to help the client.	* … I hope you know you can allow yourself to be completely honest with me here. You don’t have to be perfect for me to be in your corner … *
Empathy (EMP)	The capacity to respond with an expressed understanding of the subjective experiences of the client, including the communication of an accurate comprehension of the thoughts and emotions expresses by the client.	* … it sounds like that relationship meant a very great deal to you and there's a part of you that wants it back even though another part is scared … *
Alliance-Bond Capacity (ABC)	The capacity to create and maintain a collaborative environment wherein there is a recognition of the need to work with the client jointly on problems.	* … and that's absolutely something that you and I can work on together going forward … *
Alliance Rupture Repair Responsiveness (ARRR)	Appropriate responsiveness to a relational dynamic that either explicitly or implicitly involves some interpersonal issue which has the potential to hinder therapeutic progress.	* … Your feelings about my not getting back to you last night are completely valid and understandable given the evening you had … *

### Measures

**Figure 1:**
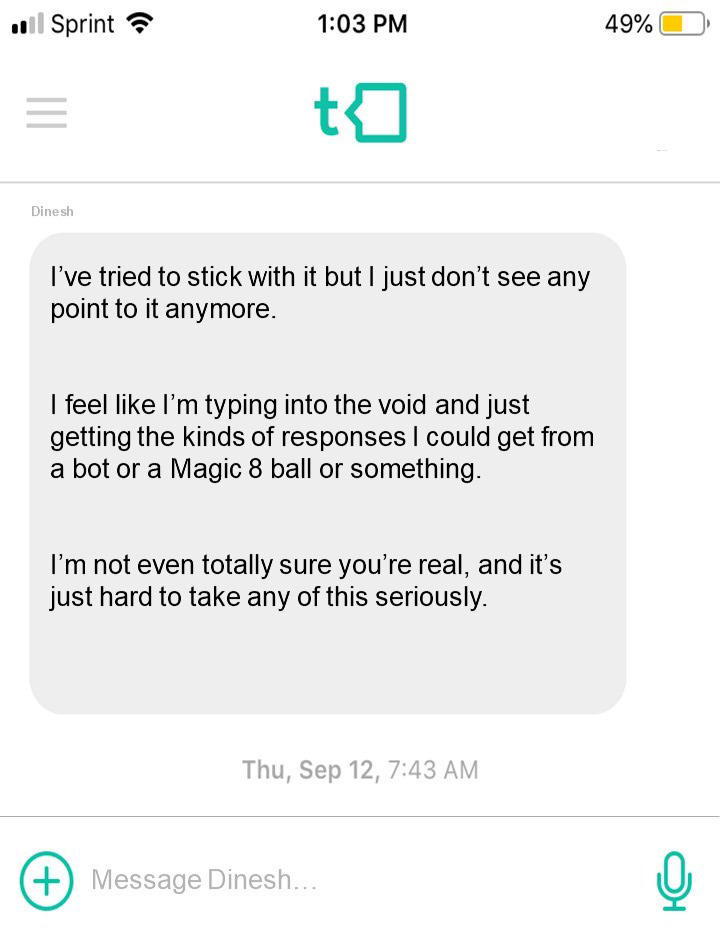
An example vignette from the FIS-T performance task. The FIS-T Task-taker is shown this message and then writes an appropriate response.

*Facilitative Interpersonal Skills —Text Performance Task and Observer Rating System (FIS-T)*: The FIS-T is an observer-report measure of therapist facilitative interpersonal skills as demonstrated through therapists' responses to eight text-message stimuli (see Figure 1 for an example stimulus). The FIS-T measures eight distinct facilitative interpersonal skills: hope and positive expectations (HPE), warmth, acceptance, and understanding (WAU), empathy (EMP), alliance-bond capacity (ABC), and alliance rupture repair responsiveness (ARRR), medium sensitivity (MS), Persuasiveness (PER), and emotional engagement (Een). The first six of the skills listed were adapted from the original FIS task, whereas the last two—MS and Een—were included to measure therapeutic processes thought to be particularly pertinent to the messaging therapy medium. Each task-taking therapist's response on the FIS-T performance task is scored by trained observers on these eight dimensions using a 5-point Likert scale ranging from 1 (“skill deficit”) to 5 (“optimal presence of skill”). All ratings are initially set at 3 (“neutral”), and are adjusted up or down based on various features of the response being coded, as outlined in the coding manual. Half scores (e.g., 1.5, 2.5, 3.5, and 4.5) are permitted if a given response falls between two anchor scores on the Likert scale. Item scores can then be averaged to create an overall mean FIS-T score per task-taker. In a previous analysis of the FIS-T Performance Task, the text-based FIS-T stimuli were previously shown to present task-taking therapists with a similarly broad range of interpersonal dynamics and perceived response difficulties compared to the original video-based FIS stimuli. More thorough description on the development and validation of the FIS-T Performance Task and Rating System is detailed elsewhere (21).

### Model development

Because therapists' text responses are scored along a 1–5 scale, the core algorithmic task was regression (i.e., scoring along a continuum) rather than classification (e.g., indicating whether a response should be categorized as “high empathy” or “low empathy”). The formal task is then to use provider FIS-T text responses to predict human-generated subdomain scores (e.g., warmth, empathy) and overall FIS-T scores. Two machine learning techniques were deployed: a Support Vector Regressor (SVR) and a Bidirectional Encoder Representations from Transformers (BERT).

SVRs are a common statistical model that have shown robust performance in a variety of classification and regression tasks in the growing field of psychotherapy natural language processing (25, 26). Our SVR algorithm was built using the Python-based SciKit-Learn machine learning library (27). First, all therapist messages were preprocessed to remove punctuation, make every letter lowercase, and remove numbers. We then converted each message into a series of unigrams (i.e., a single word as a unique predictor) and bigrams (i.e., two-word sets). We used the term frequency-inverse document frequency (Tf-idf) method to weigh these unigrams and bigrams (28). Tf-idf is an established NLP method that gives more importance to less commonly-used words (e.g., “devastated”, “indubitably”) versus common words (e.g., “the”, “and”). SVR models tend to learn more efficiently with fewer, more salient predictors. Accordingly, we used singular value decomposition (SVD) to reduce the dimension of the tf-idf vector to 75 dimensions. These 75 dimensions form a representation of the dataset composed of orthogonal vectors. We then trained an SVR model with linear kernel, C parameter of 3, and epsilon of 0.1 taking 5 s on an Intel i7-11700. 5-fold cross validation was used for selecting hyperparameters and final evaluation.

The second machine learning algorithm we used was BERT–a deep-learning technique that incorporates attentional mechanisms (i.e., Transformers) to model the contextual relations between words (29). Since its initial development, several iterations of BERT have been developed to serve different use cases. We chose to deploy DistilBERT, a less computationally-intensive BERT algorithm which has been shown to retain 97% of BERT's language understanding capabilities while running 60% faster (30). Our version of DistilBERT was downloaded from the HuggingFace transformers library (31). This model was trained on an NVIDIA 3,070 and took approximately 18 h to train. Just as with our SVR, 5-fold cross validation was used for selecting hyperparameters and final evaluation.

### Model evaluation

The SVR and DistilBERT's automated FIS-T scoring performance was evaluated against the “ground-truth” of human ratings. In other words, we evaluated these algorithms' performance in terms of how accurate their ratings are compared to those of human raters. Three performance metrics were computed for both the overall FIS-T score as well as each FIS-T subdomain:

- **R-squared:** The proportion of variance in the human FIS-T scores that can be explained by the algorithmically-generated FIS-T scores. A higher R-squared value indicates better alignment between human raters and the algorithm (i.e., more algorithmic accuracy).
- **Mean absolute error (MAE):** The average absolute difference between the actual (human-coded) and predicted (algorithmically generated) FIS-T scores. A lower MAE indicates greater algorithmic accuracy.
- **Root mean square error (RMSE):** The root of the average squared difference between actual and predicted FIS-T scores. Relative to MAE, RMSE penalizes algorithms more for making larger prediction errors. A lower RMSE indicates greater algorithmic accuracy.

## Results

### Therapist demographics

Table 2 summarizes key descriptive statistics related to our sample of task-taking therapists. Of the 978 task takers, 81.6% (*n *= 798) were women. This sample endorsed a wide range of theoretical orientations, with the most prevalent being 2nd Wave Cognitive-Behavioral (*n *= 314; 32.1%), Person-centered (*n *= 288; 29.4%), and 3rd Wave Cognitive-Behavioral (e.g., ACT, DBT) (*n *= 199; 20.3%). The majority of therapists had at least three years of experience providing therapy and a significant minority had 11 or more years of experience (*n *= 321, 32.8%). However, most therapists had much less experience providing messaging therapy; 818 (83.6%) had between zero and two years of messaging therapy experience.

**Table 2:** Demographics of participating therapists (*n* = 978).

	*n*	%
**Gender**		
Male	798	81.6%
Female	180	18.4%
**Primary Theoretical Orientation**		
2nd Wave Cognitive/Cognitive Behavioral	314	32.1%
Person-centered/Supportive/Rogerian	288	29.4%
3rd Wave Cognitive-Behavioral (ACT, DBT, etc)	199	20.3%
Psychodynamic/Psychoanalytic	79	8.1%
Existential/Phenomenological/Humanistic	41	4.2%
Experiential/Emotion-Focused (EFT, AEDP, STDP)	30	3.1%
Interpersonal (IPT)	27	2.8%
**Therapy Experience**		
0–2 years	192	19.6%
3–5 years	232	23.7%
6–10 years	233	23.8%
11+ years	321	32.8%
**Messaging Therapy Experience**		
0–2 years	818	83.6%
3–5 years	97	9.9%
6–10 years	40	4.1%
11+ years	23	2.4%

### Score distributions

**Figure 2:**
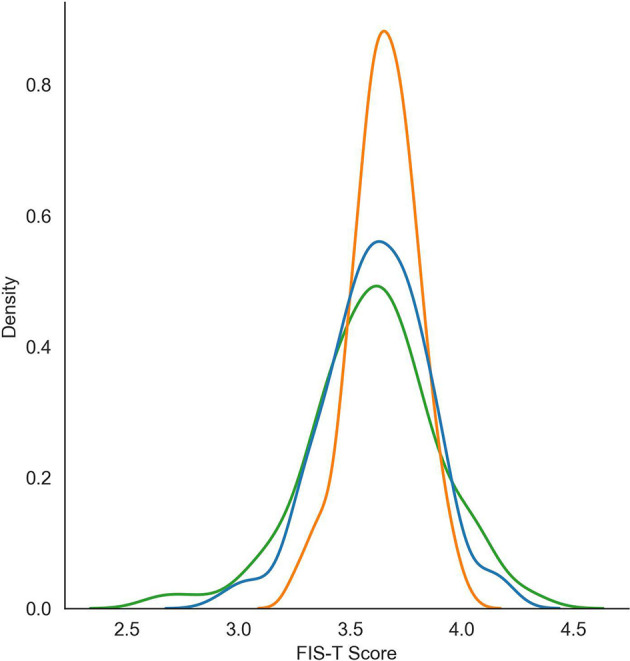
FIS-T score distributions for the DistilBERT (BERT) algorithm, support vector regressor (SVR), and ground truth (human) raters.

A distribution of human- and algorithmically-generated overall FIS-T scores are reported in Figure 2. Although the cross-validation mean overall FIS-T score was similar across humans (mean = 3.61), SVR (mean = 3.65), and DistilBERT (mean = 3.62), there was a significant difference in the ways that scores were distributed. Notably, the standard deviation of ratings generated by DistilBERT (SD = 0.230) were much closer to the distribution of human ratings (SD = 0.308) versus that of the SVR (SD = 0.146). As shown in Figure 2, DistilBERT's FIS-T scoring distribution bore a much closer resemblance to the score distribution of human raters when compared to the SVR, with the SVR's predicted scores clustered much more closely to the mean. Functionally, this bias towards the mean limits the ability of the SVR to detect outlier performance. All else equal, a scoring algorithm that provides a ratings distribution closer to the ground truth of human coders (in this case, DistilBERT) is preferable to one that biases its results towards the mean.

### Model performance metrics

Table 3 reports the three principal performance metrics used to evaluate our DistilBERT and SVR models. As previously noted, *higher* R-squared scores, *lower* MAE scores, and *lower* RMSE scores all indicate better model performance. The predictive performance of both the DistilBERT and the SVR model varied widely by FIS-T dimension. Both DistilBERT and SVR performed best when predicting total FIS-T score and the Alliance Bond dimension, whereas both models realized relatively low performance when predicting Hope, Persuasiveness, and Medium Sensitivity.

**Table 3:** R-squared, MAE, and RMSE values for SVM and BERT for each FIST subdomain.

**FIS-T Domain**	**SVR**			**DistilBERT**		
	*R-Squared*	*MAE*	*RMSE*	*R-Squared*	*MAE*	*RMSE*
Medium Sensitivity	0.136	0.250	0.312	0.238	0.229	0.294
Hope	0.259	0.235	0.297	0.251	0.236	0.299
Persuasiveness	0.390	0.249	0.317	0.364	0.255	0.324
Emotional Engagement	0.233	0.247	0.307	0.262	0.240	0.299
Warmth	0.294	0.230	0.299	0.361	0.218	0.283
Empathy	0.406	0.241	0.308	0.458	0.230	0.292
Alliance Bond	0.454	0.185	0.251	0.512	0.177	0.237
Alliance Rupture	0.371	0.245	0.330	0.453	0.234	0.310
Total FIS-T Score	0.444	0.162	0.212	0.588	0.134	0.186

Note: bold indicates superior relative performance versus the competing algorithm

DistilBERT outperformed the SVR model in predicting total FIS-T scores, as indicated by higher R-squared values and marginally lower MAE and RMSE values. Further, DistilBERT outperformed SVR along the R-squared performance metric (DistilBERT *R*^2^ = 0.588; SVR *R*^2^ = 0.444). DistilBERT also outperformed the SVR model across all three performance metrics in six out of eight FIS-T dimensions, with the SVR performing marginally better in the dimensions of hope (*R*^2 ^= 0.259; MAE = 0.235; RMSE = 0.297) and persuasiveness (*R*^2 ^= 0.390; MAE = 0.249; RMSE = 0.317).

### Model explainability

Beyond their performance metrics, both DistilBERT and the SVR model offer an exploratory look at the potential markers of interpersonally effective messages. Namely, the SVR model can be used to generate two lists of words (i.e., unigrams) which are most positively and negatively associated with FIS-T scores. The ten unigrams most positively associated with high FIS-T scores were: “forward”, “conversations”, “success”, “feel”, “look”, “will”, “completely”, “understanding”, “therapeutic”, and “appreciate”. The ten unigrams associated most closely with lower FIS-T scores were: “control”, “details”, “misunderstood”, “concerned”, “perceptive”, “sharp”, “text”, “conflicts”, “type”, and “alone”. As machine learning models can have hundreds or thousands of predictors, it would be overly reductive to interpret this as simply a list of the best and worst words to use in messaging therapy. In practice, we cannot interpret any of these words outside of their particular context.

The activations in DistilBERT offer another method for estimating the predictive importance of words within the context of a given message. Janizek et al. recently showed that the context interactions can be accounted for using the hessian of activation gradients. We can infer overall word importance using the average of message level importance (32). Using this technique, the ten words most predictive of higher FIS-T performance were: “Sounds”, “Great”, “Bit”, “Feels”, “Very”, “Wonder”, “Important”, “Lot”, “Completely”, “Little”. The ten words most predictive of lower FIS-T performance were: “Decisions”, “Therapist”, “Marriage”, “Questions”, “Achieve”, “Focus”, “Results”, “Identify”, “Step”, “Ok”. This shows an interesting pattern as the positive words are a combination of affirmative words and modifiers that reflect bids for collaboration and clarification through a softening of language, while negative words may insinuate a focus on outcomes and performance.

## Discussion

In the present study, we assessed two machine learning techniques designed to score text-based responses to the Facilitative Interpersonal Skills Task for Text (FIS-T). To our knowledge, this is the first study that deploys a machine learning algorithm to assess common factors in messaging therapy. Both models performed well along our predetermined performance metrics, with DistilBERT realizing slightly higher performance in terms of MAE and RMSE, and moderately higher performance in terms of R-squared. With an overall R-squared of 0.588, the scores for our DistilBERT model were able to capture ∼58.8% of the variance in human-generated scores. Perhaps more intuitively, DistilBERT's mean absolute error of 0.134 indicates that the average score it provided was just 0.134 points away on a 1–5 scale from the score provided by a human rater (e.g., if a human rating was 3.75, the DistilBERT rating would be between 3.62 and 3.88). Finally, the DistilBERT stands out as the superior algorithm because it provided a score distribution that was closer to that of our human coders, whereas the SVR's score distribution was heavily biased towards the mean, which shows the importance of using multiple measures of algorithmic performance. If we had not used the R-squared performance metric and if we had not analyzed the models' score distributions, we might have erroneously concluded that the SVR model and DistilBERT had essentially equivalent performance. DistilBERT was more computationally intensive, taking 18 h to train versus just several minutes for the SVR model. Yet our results suggest that this computational investment can lead to materially better model performance.

It is useful to evaluate these findings in the context of other recent work at the intersection of NLP and psychotherapy process-outcome research. In a recent study, Goldberg et al. (2020) also developed an automatic FIS Task scoring algorithm using transcripts and manually scored responses of 164 video-taped FIS Task respondents. Goldberg et al.'s elastic net model was able to achieve scoring reliability between 31% and 60% of human coders, with overall score reliability that was 52% of that found among human coders. To our knowledge, this has been the only other study to date to employ NLP to automatically score a therapist performance task. A direct comparison of model performance across these two studies is impossible for several reasons, the most salient of which is that while Goldberg et al. (2020) used transcript data from the original FIS Task, the present study employed a version of the task developed specifically for message-based care. Additionally, several key performance measures in Goldberg et al. could not be computed in the present study due to our single-rater coding procedure. However, when considering one core performance metric that these two analyses shared—R-squared—the present study represents a marked improvement, with our leading DistilBERT model yielding an R-squared of 0.58, versus an R-squared of 0.19 obtained by Goldberg et al.'s elastic net regression. The performance increase in the present study could be attributed to (1) a larger dataset, (2) a BERT-based model, and (3) a text-based behavioral task that may be naturally better-suited for NLP applications.

The promising results of the present study indicate that NLP demonstrates a twofold promise. First, NLP presents as a novel psychotherapy research method. In our view, a significant driver of the research-practice divide has been the longstanding inability of researchers to gather and analyze data on therapeutic interactions as they occur moment-by-moment. By isolating the linguistic markers of particularly (in)effective messages, NLP can help us build a “bottom-up” understanding of key therapeutic constructs with historically fuzzy conceptual boundaries (33). Second, NLP can function as a central component of future provider selection and training tools. Considered without automated scoring algorithms, behavioral tasks are prohibitively costly to regularly administer and score in most practice settings. Yet if algorithms can reliably stand in for human raters, these tasks can be more feasibly integrated into provider training and evaluation, as well as self-guided deliberate practice (34, 35).

### Limitations

Both the present study and the wider NLP research method are subject to several limitations. Concerns over algorithmic bias have been voiced in a wide range of research and implementation settings, including legal sentencing (36) and facial recognition (37). In our study procedure, a degree of algorithmic bias could have been introduced by relying on single scores from human raters. While previous research suggests that these raters had good inter-rater reliability (21), it is unknown how much drift has occurred. Additionally, our sample of task-taking therapists was largely white and mostly female; it is possible that a more diverse ethnocultural mix of respondents would have led to a broader range of textual responses. As a further limitation, these therapist responses were derived from a performance task rather than a real-life therapeutic context; it is possible that there are systematic differences in the ways that messaging therapists respond in a test context as compared to a treatment delivery as usual. It should also be noted that neither of our algorithms were optimized to allow for multiple therapist messaging styles which might be equally effective at employing facilitative interpersonal skills using very different linguistic techniques. In addition, our keyword results could also be artifacts from pretraining the model. The most predictive n-grams are all common in speech and might have been selected due to their relevance to many types of speech rather than their authentic connection with the dimensions of facilitative interpersonal skill that they should, in theory, indicate. There are still limitations in the interpretability of the model and we leave it to future work to find clinically relevant associations between specific language structures and performance on the FIS-T. Lastly, it is important to note that making inferences on the markers of therapist skills in face-to-face therapy based on the present findings would be inappropriate. On its face, digital messaging therapy is a qualitatively different context versus the face-to-face therapeutic environment. Accordingly, it seems likely to us that the most relevant therapist skills would vary over these two contexts.

### Future directions

Future work involves evaluating the extent to which these FIS-T ratings relate to therapeutic outcomes, including treatment completion and symptom improvement. This work could also be extended by either training or applying our existing DistilBERT algorithm to messages gathered from a more ethnically, culturally, and sexually diverse provider population. Training language models on context-specific corpora can enhance algorithmic performance (38). Accordingly, to further enhance model performance, our leading DistilBERT could also employ a language model trained specifically on psychotherapy transcripts, rather than internet conversations. Lastly, it may be possible to apply these scoring algorithms on therapist responses to *actual* clients generated over the course of message-based care. It is critical to note that the algorithms described in the present study can only be used to assess facilitative interpersonal skills as they manifest in a text-based behavioral task, not as they arise over the course of message-based psychotherapy as such.

## Conclusion

The present study applied two NLP models to automatically score provider responses on the Facilitative Interpersonal Skills Task for Text (FIS-T), a novel assessment of core psychotherapy process measures (e.g., warmth, persuasiveness) as they arise in message-based care. Our results indicate that a DistilBERT model realizes superior performance in this paradigm relative to an SVR. More broadly, this work demonstrates how NLP can be applied as both a psychotherapy research method as well as a potential therapist training and assessment tool. Limitations notwithstanding, this technology presents a critical opportunity for researchers to analyze therapeutic conversations in depth. By analyzing at the interaction-by-interaction level, we can arrive at higher resolution answers to Gordon Paul's still relevant question: “What treatment, by whom, is most effective for this individual with that specific problem, and under which set of circumstances?” (39).

## Data Availability

The raw data supporting the conclusions of this article will be made available by the authors, without undue reservation.

## References

[1] Przybylko G Morton DP Renfrew ME. Addressing the COVID-19 mental health crisis: a perspective on using interdisciplinary universal interventions. Front Psychol. (2021) 12:644337. 10.3389/fpsyg.2021.644337 33927669 PMC8076681

[2] Rudd BN Beidas RS. Digital mental health: the answer to the global mental health crisis? JMIR Ment Health. (2020) 7(6):e18472. 10.2196/18472 32484445 PMC7298632

[3] DellaCrosse M Mahan K Hull TD. The effect of messaging therapy for depression and anxiety on employee productivity. J Technol Behav Sci. (2019) 4(1):1–5. 10.1007/s41347-018-0064-4

[4] Hull TD Malgaroli M Connolly PS Feuerstein S Simon NM. Two-way messaging therapy for depression and anxiety: longitudinal response trajectories. BMC Psychiatry. (2020) 20(1):297. 10.1186/s12888-020-02721-x 32532225 PMC7291694

[5] Wiltsey Stirman S Song J Hull TD Resick PA. Open trial of an adaptation of cognitive processing therapy for message-based delivery. Technol Mind Behav. (2021) 2(1). 10.1037/tmb0000016 (cited February 23, 2022).35369392

[6] Ewbank MP Cummins R Tablan V Bateup S Catarino A Martin AJ Quantifying the association between psychotherapy content and clinical outcomes using deep learning. JAMA Psychiatry. (2020) 77(1):35–43. 10.1001/jamapsychiatry.2019.2664 31436785 PMC6707006

[7] Barkham M Lutz W Lambert MJ Saxon D. Therapist effects, effective therapists, and the law of variability. In: Castonguay LG Hill CE, editors. How and why are some therapists better than others?: understanding therapist effects. Washington: American Psychological Association (2017), p. 13–36. Available at: http://content.apa.org/books/16004-001 (cited February 23, 2022).

[8] Johns RG Barkham M Kellett S Saxon D. A systematic review of therapist effects: a critical narrative update and refinement to review. Clin Psychol Rev. (2019) 67:78–93. 10.1016/j.cpr.2018.08.004 30442478

[9] Wampold BE Bolt DM. Therapist effects: clever ways to make them (and everything else) disappear. Psychother Res. (2006) 16(2):184–7. 10.1080/10503300500265181

[10] Lambert MJ. Bergin and Garfield’s handbook of psychotherapy and behavior change. New York, NY: John Wiley & Sons (2013).

[11] Saxon D Barkham M. Patterns of therapist variability: therapist effects and the contribution of patient severity and risk. J Consult Clin Psychol. (2012) 80(4):535. 10.1037/a0028898 22663902

[12] Frank JD Frank JB. Persuasion and Healing: a Comparative Study of Psychotherapy. Baltimore, MD: JHU Press (1993). 376.

[13] Geller SM Greenberg LS. Therapeutic presence: A mindful approach to effective therapy. Washington, DC: American Psychological Association (2012).

[14] Stiles WB Horvath AO. Appropriate responsiveness as a contribution to therapist effects. In L. G. Castonguay & C. E. Hill (Eds.), *How and why are some therapists better than others?: Understanding therapist effects*. American Psychological Association (2017). pp. 71–84.

[15] Rogers CR. On becoming a person: A therapist’s view of psychotherapy. Boston, MA: Houghton Mifflin (1961).

[16] Wampold BE Imel ZE. The great psychotherapy debate: The evidence for what makes psychotherapy work. New York, NY: Routledge (2015).

[17] Cuijpers P Reijnders M Huibers MJ. The role of common factors in psychotherapy outcomes. Annu Rev Clin Psychol. (2019) 15:207–31. 10.1146/annurev-clinpsy-050718-095424 30550721

[18] Anderson T Ogles BM Patterson CL Lambert MJ Vermeersch DA. Therapist effects: facilitative interpersonal skills as a predictor of therapist success. J Clin Psychol. (2009) 65(7):755–68. 10.1002/jclp.20583 19437509

[19] Anderson T Crowley MEJ Himawan L Holmberg JK Uhlin BD. Therapist facilitative interpersonal skills and training status: a randomized clinical trial on alliance and outcome. Psychother Res. (2016) 26(5):511–29. 10.1080/10503307.2015.1049671 26344392

[20] Heinonen E Nissen-Lie HA. The professional and personal characteristics of effective psychotherapists: a systematic review. Psychother Res. (2020) 30(4):417–32. 10.1080/10503307.2019.1620366 31122157

[21] Foley K Hull TD Zech J Anderson T. Assessing reatment Quality in Digital Messaging Therapy: the Development and Validation of a Text-Based Facilitative Interpersonal Skills Task. New York: Talkspace, Inc. (2022). Available at: https://osf.io/4z98f/

[22] Aafjes-van Doorn K Kamsteeg C Bate J Aafjes M. A scoping review of machine learning in psychotherapy research. Psychother Res. (2021) 31(1):92–116. 10.1080/10503307.2020.1808729 32862761

[23] Atkins DC Rubin TN Steyvers M Doeden MA Baucom BR Christensen A. Topic models: a novel method for modeling couple and family text data. J Fam Psychol. (2012) 26(5):816. 10.1037/a0029607 22888778 PMC3468715

[24] Goldberg SB Tanana M Imel ZE Atkins DC Hill CE Anderson T. Can a computer detect interpersonal skills? Using machine learning to scale up the facilitative interpersonal skills task. Psychother Res. (2021) 31(3):281–8. 10.1080/10503307.2020.1741047 32172682 PMC7492408

[25] Arevian AC Bone D Malandrakis N Martinez VR Wells KB Miklowitz DJ Clinical state tracking in serious mental illness through computational analysis of speech. Scilingo EP, editor. PLoS One. (2020) 15(1):e0225695. 10.1371/journal.pone.0225695 31940347 PMC6961853

[26] Idalski Carcone A Hasan M Alexander GL Dong M Eggly S Brogan Hartlieb K Developing machine learning models for behavioral coding. J Pediatr Psychol. (2019) 44(3):289–99. 10.1093/jpepsy/jsy113 30698755 PMC6415657

[27] Pedregosa F Varoquaux G Gramfort A Michel V Thirion B Grisel O Scikit-learn: machine learning in python (2011). 6.

[28] Ramos J. Using tf-idf to determine word relevance in document queries. *InProceedings of the first instructional conference on machine learning*; (2003). p. 29–48.

[29] Devlin J Chang MW Lee K Toutanova K. BERT: Pre-training of deep bidirectional transformers for language understanding. arXiv:181004805 [cs] (2019). Available at: http://arxiv.org/abs/1810.04805 (cited February 23, 2022).

[30] Sanh V Debut L Chaumond J Wolf T. DistilBERT, a distilled version of BERT: smaller, faster, cheaper and lighter. arXiv:191001108 [cs] (2020). Available at: http://arxiv.org/abs/1910.01108 (cited February 23, 2022).

[31] Wolf T Debut L Sanh V Chaumond J Delangue C Moi A HuggingFace’s transformers: State-of-the-art natural language processing. arXiv:191003771 [cs] (2020). Available at: http://arxiv.org/abs/1910.03771 (cited February 23, 2022).

[32] Janizek JD Sturmfels P Lee SI. Explaining explanations: Axiomatic feature interactions for deep networks. arXiv:200204138 [cs, stat] (2020). Available at: http://arxiv.org/abs/2002.04138 (cited February 23, 2022).

[33] Mulder R Murray G Rucklidge J. Common versus specific factors in psychotherapy: opening the black box. Lancet Psychiatry. (2017) 4(12):953–62. 10.1016/S2215-0366(17)30100-1 28689019

[34] McLeod J. How students use deliberate practice during the first stage of counsellor training. Couns Psychother Res. (2022) 22(1):207–18. 10.1002/capr.12397

[35] Rousmaniere T Goodyear RK Miller SD Wampold BE. The cycle of excellence: Using deliberate practice to improve supervision and training. Hoboken, NJ: John Wiley & Sons (2017).

[36] Završnik A. Criminal justice, artificial intelligence systems, and human rights. ERA Forum. (2020) 20(4):567–83. 10.1007/s12027-020-00602-0

[37] Lee NT Resnick P Barton G. Algorithmic bias detection and mitigation: Best practices and policies to reduce consumer harms. Brookings (2019). Available at: https://www.brookings.edu/research/algorithmic-bias-detection-and-mitigation-best-practices-and-policies-to-reduce-consumer-harms/ (cited February 23, 2022).

[38] Alsentzer E Murphy JR Boag W Weng WH Jin D Naumann T Publicly available clinical BERT embeddings. arXiv:190403323 [cs] (2019). Available at: http://arxiv.org/abs/1904.03323 (cited October 11, 2021).

[39] Paul GL. Strategy of outcome research in psychotherapy. J Consult Psychol. (1967) 31(2):109. 10.1037/h0024436 5342732

